# Effect of plyometric training based on the integral reactive strength index of national-level jumpers and sprinters. A randomized crossover trial

**DOI:** 10.5114/biolsport.2026.152351

**Published:** 2025-08-06

**Authors:** Raynier Montoro-Bombú, Hugo Sarmento, Armando Costa, Valter Pinheiro, Paulo Malico Sousa, Carlo Buzzichelli, Luis Rama

**Affiliations:** 1 University of Coimbra, Faculty of Sport Sciences and Physical Education, 3040-256 Coimbra, Portugal; 2 CIPER, University of Lisbon, Faculty of Human Kinetics, Cruz Quebrada, Dafundo, Portugal; 3 ISCE—Polytechnic University of Lisbon and Tagus Valey, Department of Sport Sciences, 2620-379 Lisbon, Portugal; 4 Live Quatity Research Center (LQRC), Complexo Andaluz, Apartado 279, 2001-904, Santarém, Portugal; 5 University of Sport Sciences (UCCFD). Manuel Fajardo, 10600, Havana, Cuba

**Keywords:** Intensity, Volume, Reactive Capacity, Drop Jump, Jump training program

## Abstract

The Integral Reactive Strength Index (IRSI) has recently been described in the literature as a new indicator that normalizes the reactive strength index based on fall height. This study aimed to compare the effects of a plyometric training program based on IRSI versus a traditional jump training program on spatiotemporal variables associated with the performance of national-level jumpers and sprinters. A randomized, crossover trial with three parallel groups was conducted. Twenty-seven male track and field athletes were divided into three groups. The G-RT1 group consisting of 9 participants, began the experiment with IRSI-based training as the load prescription criterion. The G-PT2 group consisting of 10 participants, started the experiment with traditional plyometric training, and the control group, G-CT3, consisting of 8 participants, continued its usual training. After 19 weeks, a two-way mixed analysis of variance for the G-RT1 group found a significant interaction and large effect sizes (ES) in the group-time relationship in the 60 m dash (p ≤ 0. 001; f = 101.2; η^2^ = 0.089), and the standing triple jump (STJ) (p ≤ 0.001; f = 119.8; η^2^ = 0.031). The G-PT2 also showed better results, but the ES was lower for the standing long jump (p ≤ 0.001; f = 52.7; η^2^ = 0.045), and the STJ (p ≤ 0.001; f = 22.7; ηp^2^ = 0.011). Meanwhile, the G-CT3 did not show significant improvement at the end of the program. It is concluded that the IRSI-based plyometric training program produces better results than the traditional program. The use of IRSI-based improves individualization, emphasizes high-intensity maintenance, and induces better results with less training volume.

## INTRODUCTION

The force applied during rapid movements is crucial for athletic performance, representing the external expression of muscular tension and reflecting a dynamic maximal force capacity [1]. Hence, researchers are concerned about measuring the maximal force in the shortest possible time [2]. It has also been pointed out that in most athletic events, it is more critical to apply maximal force quickly than to have only a high level of maximal force [3]. This is because the most representative actions in sports have ground contact times (GCT) of less than 250 ms [4]. These insights suggest that modern training models for sprinters or jumpers should focus on preparing athletes to apply force quickly rather than emphasizing hypertrophy or using high loads at low velocities. Previous findings [5, 6] have shown that the best way to transfer strength to a sport-specific action is through exercises that share similar force-time characteristics with those movements (e.g., similar rate of force development, power, time to force peak, impulse). The plyometric training programs (PTP), due to their inherent characteristics, are designed to generate large reaction forces by getting proprioceptive signals processed by the central nervous system to control muscle length and tension, resulting in the stretch-shortening cycle (SSC), that releases the elastic energy accumulated during the propulsion phase [7]. Therefore, PTP could be considered one of the most effective ways to train force when applied in fast dynamic movements that require a high magnitude of force with minimal contact times.

Researchers [8–11] have addressed the importance of jump training programs (JTP). However, it is crucial to distinguish these from plyometric training programs (PTP). The key difference lies in the variety of jumps included in the JTPs, whereas PTPs are exclusively focused on plyometric jumps. Currently, we can find proposals of JTP for the development of the running speed [12–14], changes of direction [13, 15], reactive strength [16, 17], and power [14, 18]. Others also considered the different types of landing surfaces [19, 20], the use of bilateral and unilateral jumps [21], and how the volume variation affects jumping performance [22]. The latter provided better results when comparing the single with the combined JTP on jump kinetics and kinematics [23]. Others found that a unilateral JTP showed higher improvement in strength and rate of force development for each leg individually [21] and that short-term JTP using different volumes and landing surfaces can improve explosive performance [19].

On the other hand, seeking to identify the effectiveness of high and low-volume plyometric loads on the stretch-shortening cycle, researchers [22] have reported that a low-volume JTP produces the same improvement in RSI performance as a high-volume program. Undoubtedly, the best way to develop plyometric activities is through the correct organization of a program. While various JTPs have been explored, a consistent issue is the lack of individualized volume and intensity prescription based on reactive strength capabilities. This is in line with a recent systematic review showing that most of the studies presented methodological design problems, thus reporting inconsistent volume and intensity parameters [24].

Recent research has shown that the reactive strength index (RSI) presents challenges in its interpretation and practical implementation [25]. This research found that increasing height fall (HF) from 0.30 cm to 0.50 cm did not produce significant differences in the RSI result. This finding raised concerns, as previous studies have demonstrated that increasing HF enhances neuromuscular pre-activation levels and the velocity achieved during the eccentric phase, thereby augmenting the contractile potentiation mechanisms of muscles and tendons [26]. In this context, accepting that reactive strength remains constant or relatively constant as HF increases may be implausible. Similarly, two athletes jumping from the same HF may not be exposed to the same relative intensity [13]. Also, the RSI guides us to stimulate reactive strength in previously adapted areas rather than the regions that could exhibit greater specificity for high-performance athletes [16]. For these reasons, the researchers propose the integral reactive strength index (IRSI) as a metric that considers the athlete’s reactive strength in accordance with the FH [25].

If these assumptions are correct, the IRSI contribute to the individualization process since athletes would be training based on their characteristics rather than a predetermined volume of sets and repetitions, sometimes presenting poor scientific support. Although this approach seems interesting, does PTP based on the IRSI as a volume and intensity prescription criterion provide better results than traditional? No scientific report has been published to date comparing both programs. There are currently no studies comparing how PTP based on the IRSI affects spatiotemporal variables such as standing long jump (SLJ), standing alternating triple jump (STJ), sprint, and DJ results at different FH commonly used as training strategies or training monitoring tests for sprinters and jumpers in athletics. In this context, the present study aimed to compare a PTP based on the IRSI versus a traditional PTP on the spatiotemporal variables associated with the performance of national-level jumpers and sprinters. It is hypothesized that a PTP based on the IRSI has better effects than a traditional PTP on spatiotemporal variables associated with the performance of jumpers and sprinters at the national level.

## MATERIALS AND METHODS

### The experimental approach to the Problem

**FIG. 1:**
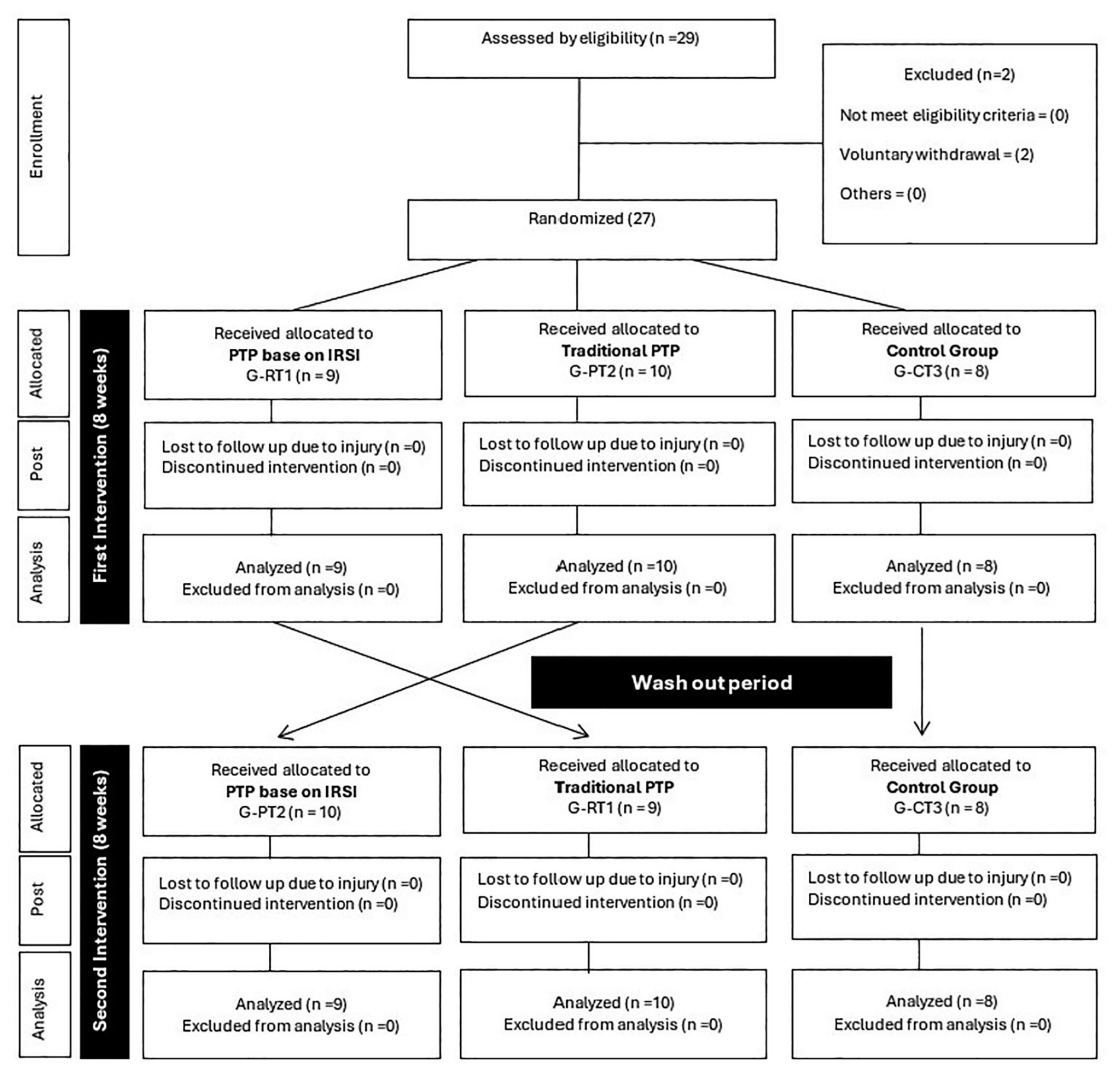
CONSORT Diagram: Participant flow at each stage of the randomized crossover trial with three parallel groups. G-RT1 = Group starting the intervention with Integral Reactive Strength Index; G-PT2 = Group starting the intervention with traditional program and G-CT3 = Control group.

To test the hypothesis that the IRSI based PTP has better effects than a traditional PTP on spatiotemporal variables associated with the performance of jumpers and sprinters at the national level, a randomized crossover trial with three parallel groups was conducted. The experiment lasted 19 weeks (Figure 1), during which the groups were randomized using an open-access tool (http://www.jerrydallal.com/random/randomize.htm). Allocation concealment was ensured to avoid bias. One group, G-RT1, started the experiment with PTP based on the IRSI. Another group, G-PT2, began the experiment with traditional PTP, while the G-CT3 was a control group and continued its regular training process (modality-specific technique training, running speed, technical jumps and strength training) without any specific plyometric intervention. The washout period for crossover between program 1 (PTP-1) and program 2 (PTP-2) was 2 weeks. The groups (G-RT1, G-PT2, and G-CT3) constituted the independent variable, while the performance variables (SLJ, 60 m, STJ, and IRSI) constituted the dependent variables.

### Participant

A total of 29 subjects were recruited, with two withdrawing voluntarily. Twenty-seven male track and field athletes (19.2 ± 3 years; height 177 ± 5 cm; body mass 71.6 ± 8.14 kg; BMI = 21.89 ± 3.14 kg m^−2^) were analyzed. For the recruitment of participants, the following inclusion criteria were established: nationallevel junior athletes, Lisbon Athletics Association members, with more than three years of experience in track and field short sprints (11.02 ± 0.18 s for 100 m) and long jump events (6.34 ± 0.31 m), with a total of approximately 16 hours of training per week and according to the previous criteria with more than 2 years of plyometric training experience [24]. The exclusion criteria were athletes who had suffered injuries in the previous six months, with orthopedic problems or medical indications, absent from the training in the previous three months. All were informed of the potential risks associated with the study participation and gave written informed consent. The research was conducted following the recommendations of the latest version of the Declaration of Helsinki (October 2013). It was approved by the Scientific Council and the Ethics Committee of the Faculty of Sport Sciences and Physical Education of the University of Coimbra (code-CE/FCDEF-UC/00802021 on July 6, 2021).

### Procedure

The experiment was carried out during the summer athletic season. The week before the start of the study was dedicated to familiarizing the athletes with the training tasks and completing the pretest assessment, during which anthropometric data were collected. Height was measured on a stadiometer with an accuracy of 0.1 cm (Bodymeter 206, SECA, Hamburg, Germany). Body mass was assessed on a scale (SECA scale, Hamburg, Germany), with an accuracy of 0.1 kg. The body mass index was determined, considering previous recommendations (29). Data were also collected for the 60 m sprint, SLJ, and STJ. In the following training session, rebound DJ data were collected at different FHs. Before the evaluation protocol, athletes completed a standard warm-up as established in previous research [25]. The warm-up included three laps of the track (400 m) and dynamic stretching. Athletes then had 30 minutes to perform an individual warm-up specific to their discipline. After 5 minutes of recovery, measurements were taken.

### 60 m sprint

It took place on an official synthetic track. All athletes wore specialized sprinting spikes. The times were recorded using photocells Witty gate (Microgate, Bolzano, Italy). Witty gate has an integrated transmission system with a range of 150 m. Redundant radio transmission ensures that the acquired data is transmitted to the timer with maximum accuracy (± 0.4 milliseconds). The Witty gate was calibrated according to the manufacturer’s recommendations in linear single-shot mode and placed 0.15 m before the starting line and 0.20 m above ground level. The second cell was standardized at a height of 1.20 m above ground. The start was standardized for all, with a three-point start position (typical of athletics). All athletes decided on their own start time, so no verbal stimulus was given to begin the test. As a result, the recorded times did not account for reaction time. The athletes performed two runs with 8 to 10 minutes apart for recovery. The best of the two attempts was registered for data analysis.

### SLJ and STJ test

The SLJ was performed in the sand pit (athletics track). All athletes wore specialized athletic spikes and stood behind a line 0.10 m away from the sand pit. With their feet shoulder-width apart, they performed a powerful horizontal push-off with free arms. The triple jump test was performed from 7.50 m from the sand pit. From a static position, with feet shoulder-width apart, athletes performed an initial power push, followed by a hop, a step, and a jump towards the sand pit (first supporting leg according to the athlete’s choice). Athletes performed two attempts of SLJ with approximately 2 min recovery and the STJ attempts with approximately 3–4 min recovery between each. After each jump, the sand was smoothed to make it flat and ready for the next attempt. The data were recorded with a 50 m metric tape measure (Metal Works, Concesio, Italy), referencing the last footprint left by the athlete in the sand. For data analysis, the best of the two jump attempts was considered.

### IRSI test

The athletes performed DJ from different FHs (0.30, 0.40, 0.50, and 0.60 m). For this purpose, they placed four boxes at the heights described above and the Optojump Nex system (Microgate, Bolzano, Italy) on both sides of the boxes at 4 m from each other and with a separation of 0.10 m between the optical system and the boxes. According to the manufacturer’s data, Optojump has an optical data acquisition system, consisting of a transmitting and a receiving optical bar. Each contains 96 infrared LEDs (1.0416 cm. resolution) with an accuracy of 1/1000 of a second. With these features, Optojump collected the data of GCT and jump height (JH) and transferred to an Excel spreadsheet to calculate the IRSI (equation 1) [25]. The values were instantly projected into a wall in front of the jump area and verbally reported by the researcher. The test was stopped when the IRSI value of the previous FH was greater than or equal to that of the current FH. If the IRSI was higher than the previous FH, the previous FH was recognized as the one with the best IRSI. Conversely, if the IRSI was equal to the previous FH, the new FH was acknowledged and recognized as having the best IRSI. All DJs were performed with bounces and the double target of minimizing GCT and maximizing JH. Recovery between attempts of the same FH was approximately 1 min, and between different FH was approximately 4 min. All DJ showed high reliability (ICC DJ30 = 0.92; DJ40 = 0.94; DJ50 = 0.90 and DJ60 = 0.87).


IRSI=RSI−RC

### Equation (1)

Where: IRSI was previously described as integral reactive strength index, RSI = Reactive strength index, and RC = reactive capacity, which is calculated from FH divided by JH.

### Training program

Each training program was conducted in parallel for eight weeks. The G-RT1 group started the experiment with the PTP-1 based on the IRSI (Table 1). For this program, training volume was determined by maintaining the intensity over 95% of the maximum FH (individualized at 0.30 and 0.50 m) achieved and the best score on the IRSI without a predetermined volume. Therefore, the number of repetitions per set was strictly individualized and depended on maintaining the intensity level. In none of the sets were athletes allowed to exceed 10 repetitions per set. The number of sets was standardized to four to establish a similarity between the number of sets of both programs. The intensity was monitored with the Optojump (Microgate, Bolzano, Italy), and each athlete was informed of the IRSI score results, including when they experienced a loss of 5%. This program followed previous recommendations for the methodological organization of plyometric training programs [24].

**TABLE 1:** Training program based on the IRSI

Exercises	FH	Intensity	Repetitions per set	Rest Intervals between reps	Sets	Rest Intervals between sets	Weekly frequency	Time between sessions
DJ	Best IRSI	95–100% maximum IRSI	Up to 5% loss of best IRSI.	50 ± 5 seg.	4	4–6 min	3	48 hours

DJ = drop jump; FH = fall in height; IRSI = integral reactive strength index; Rep = repetitions, Rec = recovery.

Traditional training (PTP-2) was based on established volume criteria [27], but eight weeks were maintained. The volume was set to four sets of 10 repetitions, and the same frequency and intra- and intersession recovery conditions proposed in Table 1 were maintained. The FH of PTP-2 was also adjusted according to its best IRSI obtained in the initial assessment.

On the third and fourth days after the end of PTP-1, the athletes performed the first post-test in the same order as the pretest. The final post-test was performed on the fourth and fifth days after the end of PTP-2. During the two assessments, the athletes performed the same warm-up described in the pretest. Then they supplemented it with their regular training (modality-specific technique training, running speed, technical jumps and strength training).

### Statistical analysis

Data are presented as mean and standard deviation (mean ± SD). Assumptions of normality and homogeneity were tested using the Shapiro-Wilk test. To compare the level of significance of the programs within the same group (group × time) and for the independent analysis (group × group), a repeated measures ANOVA (Pretest + post-test PTP-1 + post-test PTP-2) was used, using the Bonferroni post hoc test. Intra-section reliability was calculated using the ICC to check the consistency between reported data. Partial eta squared (ηp^2^) was calculated to estimate the effect size (ES) of the two-way mixed analysis of variance. ES was interpreted using the mean differences between the two independent groups described above (0.20 = small, 0.50 = medium, and 0.80 = large) [28]. Using the G*Power software (v.3.1.9.7, Heinrich-Heine University of Düsseldorf, Germany) the statistical power was established a priori (ANOVA for repeated measures, within-individual interactions). The beta value (1-β err prob) was 95%, with an alpha (α ≤ err prob) of 0.05 and an effect size of 0.4, requiring a sample size of 24 subjects. All analyses set the significance level at p < 0.05 (5%). Data analysis was performed with the statistical program Jamovi project (2022) (Version 2.3 Computer Software).

## RESULTS

At the end of PTP 1, we reported a significant increase in the grouptime interaction between the dependent variables, where: SLJ (p ≤ 0.001; f = 95.4; η^2^ = 0.048), 60 m (p ≤ 0.001; f = 101.2; η^2^ = 0.089), STJ (p ≤ 0. 001; f = 119.8; η^2^ = 0.031), DJ30 IRSI (p ≤ 0.001; f = 84.6; η^2^ = 0.145), DJ40 IRSI (p ≤ 0.001; f = 56.8; η^2^ = 0.163), DJ50 IRSI (p ≤ 0.001; f = 40.7; η^2^ = 0.068) and DJ60 IRSI (p ≤ 0.001; f = 27.8; η^2^ = 0.078). Identical results were also observed at the end of PTP 2 where SLJ (p ≤ 0.001; f = 52.7; η^2^ = 0.045), 60 m (p ≤ 0.001; f = 42.2; η^2^ = 0.055), STJ (p ≤ 0.001; f = 22.7; ηp^2^ = 0. 011), DJ30 IRSI (p ≤ 0.001; f = 29.1; η^2^ = 0.042), DJ40 IRSI (p ≤ 0.001; f = 20.45; ηp^2^ = 0.011), DJ50 IRSI (p ≤ 0.001; f = 30.9; η^2^ = 0.035) and DJ60 IRSI (p ≤ 0.001; f = 35.3; ηp^2^ = 0.050). More detailed characteristics of the group × time relationship between the programs during the pretest, before and after the crossover (p = Bonferroni, ES, 95% CI) are reported in Table 2.

**TABLE 2:** Effects of the group-time relationship between dependent variables during the pretest and between programs both before and after crossover.

Variables	Groups	Pre-Test		Test PTP 1		Test PTP 2	Effect of PTP 1			Effect of PTP 2		

							ES	IC (95%) Upper– Lower	Increase ES	ES	IC (95%) Upper– Lower	Increase ES
**SLJ (m)**	G-RT1	2.83 ± 0.129	8-week intervention G-RT1(IRSI) vs G-PT2 (Traditional)	2.91 ± 0.131	8-week intervention G-PT2 (IRSI) vs G-RT1 (Traditional)	2.96 ± 0.127	2.31^***^	3.57–1.00	Large	1.95^***^	3.06–0.78	Large
	G-PT2	2.84 ± 0.114		2.88 ± 0.104		2.97 ± 0.071	1.15^**^	1.95–0.32	Large	1.78^***^	2.78–0.74	Large
	G-CT3	2.88 ± 0.139		2.92 ± 0.133		2.93 ± 0.127	3.68^***^	5.68–1.66	Large	0.77	1.55–0.04	moderate
		
**60 m (seg)**	G-RT1	7.15 ± 0.113		7.05 ± 0.085		7.02 ± 0.180	3.26^***^	1.55–4.94	Large	1.78^***^	0.68–2.83	Large
	G-PT2	7.16 ± 0.076		7.11 ± 0.061		7.05 ± 0.145	2.28^***^	1.05–3.47	Large	2.10^***^	0.94–3.22	Large
	G-CT3	7.05 ± 0.046		7.04 ± 0.031		7.03 ± 0.537	0.49	1.55–0.04	Small	0.22	0.48–0.91	Trivial
		
**STJ (m)**	G-RT1	8.68 ± 0.340		8.87 ± 0.368		8.94 ± 0.355	3.05^***^	3.63–1.43	Large	1.89^***^	2.98–0.75	Large
	G-PT2	8.87 ± 0.254		8.81 ± 0.158		8.98 ± 0.250	2.35^***^	3.57–1.10	Large	4.02^***^	5.94–2.08	Large
	G-CT3	8.84 ± 0.279		8.86 ± 0.292		8.84 ± 0.320	0.59	1.33–0.18	Moderate	0.18	0.52–0.87	Trivial
		
**DJ 30 cm (m/s^−1^)**	G-RT1	0.44 ± 0.120		0.63 ± 0.119		0.70 ± 0.138	3.07^***^	4.66–1.44	Large	1.28^**^	2.16–0.36	Large
	G-PT2	0.59 ± 0.095		0.66 ± 0.116		0.83 ± 0.184	1.51^***^	2.41–0.56	Large	2.02^***^	3.11–3.22	Large
	G-CT3	0.51 ± 0.152		0.53 ± 0.148		0.53 ± 0.121	0.66	1.42–1.23	Moderate	0.49	0.25–1.22	Small
		
**DJ 40 cm (m/s^−1^)**	G-RT1	0.74 ± 0.111		0.96 ± 0.117		1.03 ± 0.131	3.07^***^	4.67–1.44	Large	1.28^***^	2.15–0.90	Large
	G-PT2	0.91 ± 0.108		0.98 ± 0.148		1.18 ± 0.229	1.37^**^	2.23–0.47	Large	1.94^***^	3.00–0.84	Large
	G-CT3	0.82 ± 0.156		0.83 ± 0.141		0.84 ± 0.086	0.57	1.31–0.19	Small	0.35	0.37– -1.05	Small
		
**DJ 50 cm (m/s^−1^)**	G-RT1	0.98 ± 0.132		1.18 ± 0.125		1.24 ± 0.150	2.72^***^	4.16–1.24	Large	1.16^**^	1.99–0.27	Large
	G-PT2	1.14 ± 0.159		1.22 ± 0.185		1.42 ± 0.258	0.90^**^	1.63–0.14	Large	1.79^***^	2.80–0.75	Large
	G-CT3	1.04 ± 0.190		1.04 ± 0.096		1.04 ± 0.194	0.06	0.62– -0.76	Trivial	0.35	0.37– -1.05	Small
		
**DJ 60 cm (m/s^−1^)**	G-RT1	1.03 ± 0.150		1.26 ± 0.115		1.35 ± 0.604	1.54^**^	2.50–0.53	Large	1.63^**^	2.64–0.59	Large
	G-PT2	1.20 ± 0.171		1.28 ± 0.197		1.52 ± 0.279	0.88^**^	1.61–0.13	Moderate	1.68^***^	2.65–0.68	Large
	G-CT3	1.10 ± 0.188		1.10 ± 0.123		1.10 ± 0.086	0.35	0.37– -1.05	Small	0.35	0.37– -1.05	Small

*** = < .0001;

** = < 0.03;

*= < 0.05; ES = Effect Size; IC (95%) = confidence interval; SLJ = standing long jump; STJ = standing triple jump; DJ = Drop Jump; G-RT1 = Group starting the intervention with Integral Reactive Strength Index; G-PT2 = Group starting the intervention with traditional program and G-CT3 = Control group; PTP = Plyometric Training Program. Effect size (ES) for paired comparisons was calculated as Cohen’s *d_z_*, defined as the mean difference between conditions divided by the standard deviation of the differences.

Similarly, it was found that during the pretest, there were differences between groups in 60 m (p ≤ 0.021; f = 4.61; ηp^2^ = 0.22.3). Also, DJ40 IRSI (p ≤ 0.010; f = 5.67; ηp^2^ = 0.275) it was different. Likewise, after PTP 1, significant differences were only found in 60 m (p ≤ 0.021; f = 4.61; ηp^2^ = 0.22.3), and at the culmination of PTP 2, differences were in DJ30 IRSI (p ≤ 0.003; f = 7.61; ηp^2^ = 0.398) and DJ40 IRSI (p ≤ 0.001; f = 8.98; ηp^2^ = 0.431). More detailed results of the differences between groups are shown in Table 3. Table 4 shows the tangible results of the group volume reduction of G-RT1 compared to G-PT2.

**TABLE 3:** Effects of the group-group relationship between dependent variables during the pretest and between programs both before and after crossover.

Pre-test						PTP 1				PTP 2		
		
Varia-bles	ANOVA Bonferroni	ES	CI = 95%	Diff. ES		ES	CI = 95%	Diff. ES		ES	CI = 95%	Diff. ES
		
			Lower–upper				Lower–upper				Lower–upper	
**SLJ (m)**	G-RT1 **vs** G-PT2	0.39	-0.609–1.399	Small	Intervention 8 weeks G-RT1 (IRSI) vs G-PT2 (Traditional)	0.98	0.05–.024	Large	Intervention 8 weeks G-PT2 (IRSI) vs G-RT1 (Traditional)	0.57	-0.43–.59	Medium
	G-RT1 **vs** G-CT3	-0.08	-1.120–0.944	Small		0.34	0.69–.383	Small		0.87	-0.19–.94	Large
	G-PT2 **vs** G-CT3	-0.48	-1.477–0.512	Small		0.63	1.63–.366	Medium		0.30	-0.68–.29	Small
		
**60 m (seg)**	G-RT1 **vs** G-PT2	-0.34	-1.348–0.657	Small		-1.38^**^	-2.471– -0.305	Large		-0.86	-1.89–.168	Large
	G-RT1 **vs** G-CT3	1.05	-0.023–2.138	Large		-0.02^**^	-1.05–.008	Small		-0.39	-1.43–.645	Small
	G-PT2 **vs** G-CT3	1.40^**^	-0.330–2.476	Large		1.36	0.29–.432	Large		0.47	-0.52–.464	Small
		
**SLJ (m)**	G-RT1 **vs** G-PT2	0.24	-0.756–1.243	Small		0.61	-0.39–.631	Medium		0.32	-0.67–.33	Small
	G-RT1 **vs** G-CT3	-0.26	-1.298–0.771	Small		0.40	-0.62–.447	Small		0.76	-0.29–.82	Medium
	G-PT2 **vs** G-CT3	-0.50	-1.502–0.489	Medium		-0.20	-0.20–.777	Small		0.43	-0.55–.43	Small
		
**DJ 30 cm (m/s^−1^)**	G-RT1 **vs** G-CT1	-1.17	-2.236– -0.117	Large		-2.45	-2.216– -0.101	Large		-0.83	-1.86–.191	Large
	G-PT2 **vs** G-CT3	-0.58	-1.634–0.459	Medium		0.78	-1.63–.459	Medium		1.01	-0.05–.094	Large
	G-PT2 **vs** G-CT3	0.58	-0.411–1.588	Medium		1.03	-0.42–.596	Large		1.85^**^	0.72–.991	Large
		
**DJ 40 cm (m/s^−1^)**	G-RT1 **vs** G-PT2	-1.33^*^	-2.414– -0.260	Large		-0.78	-1.17–.820	Large		-0.97	-2.01–.067	Large
	G-RT1 **vs** G-CT3	-0.48	-1.530–0.554	Small		0.90	-0.16–.963	Large		1.04	-0.03–.221	Large
	G-PT2 **vs** G-CT3	0.84	-0.168–1.866	Large		1.08	0.04–.121	Large		2.01^***^	0.85–.175	Large
		
**DJ 50 cm (m/s^−1^)**	G-RT1 **vs** G-PT2	-1.15	-2.216– -0.101	Large		-0.42	-143–.579	Small		-1.06	-2.111– -0.014	Large
	G-RT1 **vs** G-CT3	-0.58	-1.635–0.459	Medium		0.68	-0.36–.738	Medium		0.82	-0.23–.885	Small
	G-PT2 **vs** G-CT3	0.57	-0.429–1.596	Medium		1.11	0.07–152	Large		1.88^**^	0.74–.026	Large
		
**DJ 60 cm (m/s^−1^)**	G-RT1 **vs** G-PT2	-1.09	-2.146– -0.043	Large		-0.24	-2.153– -0.383	Small		-1.18	-2.294– -0.128	Large
	G-RT1 **vs** G-CT3	-0.44	-1.488–0.593	Small		0.86	-1.48–.593	Large		0.98	-0.08–.062	Large
	G-PT2 **vs** G-CT3	0.64	-0.356–1.650	Medium		1.11	-0.335–1.420	Large		2.17^***^	0.98–.363	Large

*** = < .0001;

** = < 0.03;

* = < 0.05;

ES = Effect Size; IC (95%) = confidence interval; SLJ = standing long jump; STJ = standing triple jump; DJ = Drop Jump; G-RT1 = Group starting the intervention with Integral Reactive Strength Index; G-PT2 = Group starting the intervention with traditional program and G-CT3 = Control group; PTP = Plyometric Training Program.

**TABLE 4:** Differences in training volume after the first and second intervention

Intervention	Group / week	1	2	3	4	5	6	7	8	Total volume
**First intervention**	G-PT1	66	74	92	98	109	114	115	117	785
	G-PT2	120	120	120	120	120	120	120	120	960
**Second intervention**	G-PT1	120	120	120	120	120	120	120	120	960
	G-PT2	72	79	87	94	110	116	116	118	792

G-RT1 = Group starting the intervention with Integral Reactive Strength Index; G-PT2 = Group starting the intervention with traditional program

## DISCUSSION

This study compared an IRSI-based PTP versus a traditional PTP on spatiotemporal variables associated with performance in nationallevel jumpers and sprinters. To our knowledge, this is the first study that seeks to individualize the volume and intensity of plyometric training based on reactive strength parameters. The main findings are that: I) both PTPs present significant improvements on the spatiotemporal variables analyzed (SLJ, STJ, 60 m, and DJ), and II) during PTP-1, the G-RT1 group showed higher ES than G-PT2. After the cross, the G-PT2 showed higher ES than the G-RT1 group, demonstrating the higher. Effects of the PTP based on IRSI. Relying on the ES, in general, the PTP based on IRSI induced, more pronounced effects in all variables (Table 2).

Our results were structured in a synchronous sequence not previously reported. Previous studies [29–33] only presented the overall outcome of the intervention without detailing the daily effect induced. In the G-RT1 group, during the initial sessions of IRSI-based PTP, although the athletes had prior experience in plyometric training [24], they exhibited self-confidence in the exercise execution only toward the end of the first training session. This improvement facilitated better adaptation to the FHs, as evidenced by greater consistency, maintaining over 95% intensity in the first repetitions. In the first PTP session, the G-RT1 group started with a mean of (5 ± 1) jumps per set.

It is likely due to better assimilation of the exercise and accentuation in intensity maintenance (95%), the ground contact time (GCT) was reduced by 4.2% in the subsequent 3 and 6 sessions, without affecting the jump height (JH). This result could also be explained by a predictive component activated during repetitive FH training, where the motor system adjusts the mapping between sensory information and the expected impact force produced. [34]. In this context, the organism adapts to estimate the FH, providing the necessary data to infer its duration and influencing kinematic and GRF variables [34]. It is likely that training at the optimal FH, 3 to 6 training sessions is sufficient to trigger these adaptations [35].

Between 5 and 9 sessions, we found that the athletes could maintain low GCT and high JH during 5 or 6 consecutive DJ repetitions, thus demonstrating a stabilization of these variables. This could have been the reason for the performance gains found in the IRSI, between 1.4 and 2.7% higher than the pretest. Using session-by-session monitoring of the performance variable IRSI, this study found that the time required to obtain the first changes in DJ performance was 3 weeks. This represented a shorter time than in other studies that found positive effects of DJ in the first 4 weeks of intervention [36, 37]. Between sessions 9 and 18, the athletes began to increase the volume of jumps while maintaining the intensity criteria, resulting in 8 ± 1 jumps per set. Between 9 and 17 sessions, we also reported the stabilization markers of the IRSI above 98% intensity between repetitions 3 and 7. Then, from session 18 until the end of the program, we observed a considerable increase in the volume of jumps with a mean of 9. It was observed that the athletes reached ten jumps maintaining an intensity above 98%. In this sense, all PTPs that optimize FH as an intensity criterion [24] and even standardize volume with 10 or more jumps per set [14, 20, 27, 38] could be questionable.

**FIG. 2:**
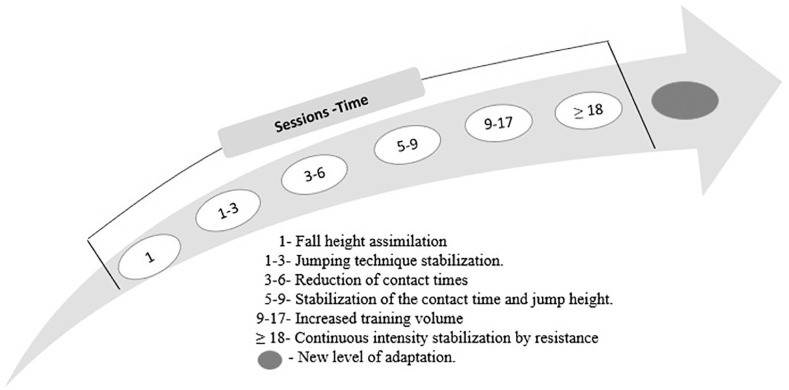
Heterochronism of plyometric training based on the IRSI

The researchers stopped the sets when they reached ten repetitions to avoid biasing the results due to increased jumps compared to the G-PT2 group. This behavior could indicate that the athletes needed to increase their individual FH, to elicit a new adaptive process [35]. These findings allow us to understand that there is a heterochronic process of the PTP based on the IRSI that is established in an individualized manner that is hardly found in programs that do not control daily performance variables [39]. Figure 2 illustrates this behavior in the analyzed athletes. It would be beneficial for future research to analyze the heterochronic behavior of power output, peak force, and other related variables.

In the G-PT2 group, which started training with a traditional program, technical execution problems were generally observed between the first 1–5 sessions, particularly after the sixth repetition. This result has not been reported previously [20, 22, 23, 40]. During G-PT2, we found visible increases in GCTs, alterations in technical execution from the rebound to countermovement [41], and the appearance of double ground contacts. Despite the constant verbal encouragement to maintain the technique pattern, this only improved progressively after training session 9. We question the PTP strategies that present similar structures [14, 27, 38] or even higher volume of sets [33, 42] and did not report technical problems in their research reports. We can understand the reasons for establishing gradual volume increments [15, 33, 43]. However, we question whether this progression is based on any interindividual criteria. At the same time, the support for the increments can be highly questionable because plyometric training requires a certain level of quality before striving for quantity. The pretest data showed that the experimental groups (G-RT1 and G-PT2) improved their performance significantly compared to the G-CT3. Nevertheless, for the DJ60, it is evident that theG-PT2 presents a higher ES (Table 2).

After the crossover, the G-RT1 group that utilized the PTP based on IRSI performed the traditional program. All athletes started with higher FHs than those established during the pretest, while among the athletes of G-PTP 2, only six changed to a higher height. When the G-PT2 worked based on the IRSI, not many problems in the jumping technique were observed. Nevertheless, at the beginning of PTP, they presented problems in maintaining low GCT. This could be a consequence of the high jumping volumes [33, 42]. For this group, the weekly progression on the maintenance of 95% intensity was not different from that previously reported in the table 2.

On the contrary, when G-RT1 increased the volume of jumps from a higher height, they presented a marked technical jump deficiency. Specifically, they increased the GCT approximately after the fourth jump and presented greater difficulty in stabilizing the technique pattern. A reduction of JH and more pronounced physical exhaustion was observed along the training program in series 3 and 4.

The SLJ after eight weeks showed significant improvements and large ES for all groups; however, we noted that, the non-intervention group that maintained their regular training process (modality-specific technique training, running speed, technical jumps and strength training) showed higher ES (Table 2). This result indicated that PTP may not be responsible for the training-associated effects after eight weeks. This finding could be due to specific characteristics included in the training of the G-CT3 that were beyond the scope of our research. In other studies, after a PTP, significant differences were found; however, they used numerous types of jumps, and it is impossible to identify which one/s had the greatest effect on the SLJ [13, 44]. Further analysis of this variable allows us to observe that after the end of the experiment (19 weeks), G-RT1 and G-PT2 achieved significant improvements and large ES compared to G-CT3. This indicates that the SLJ improvements associated with IRSI-based PTP require more than eight weeks.

The analysis of the sprint (60 m) showed that G-RT1 and G-PT2 presented significant improvements compared to the pretest (table 2). However, at the end of program 1, the IRSI-based PTP showed higher ES than traditional PTP. These results were also corroborated after cross-over 2 (table 2). It is widely recognized that in a 100-meter race, elite sprinters reach their maximum velocity between 40 and 60 meters, whereas constant acceleration during this race can be maintained up to 40 meters [45, 46]. This suggests that the 60-meter test depends on both acceleration and peak velocity. These criteria, together with the findings of previous studies, indicate that reactive jumps transfer more effectively to longer sprints than to short sprints. [32], justify the improvement observed in both groups. Even so, at the end of PTP-1, G-RT1 presented higher ES. The IRSI-based PTP may have induced improvements in the kinetic structure of movement, thus contributing to an improvement in reactive strength and decreased contact times during sprinting [47]. We could assume that the increase in power induced improvements in stride length but not in stride frequency as previously reported [48]. Our study is consistent with previously reported results [30, 49]. Research has shown that to improve the athletes’ speed, it is necessary to modify either their frequency and/or the stride length, avoiding the negative effect over each other [50, 51]. It has also been reported that exercises that improve CGT have effects on quickness, then it is not surprising that there is a positive effect of the IRSI-based PTP. It is possible that coaches and sports specialists who wish to improve stride frequency may not benefit from IRSI-based PTP and should use assisted-speed methods, as reported in previous research [31].

During multiple-jump training, coaches seek to maintain high power levels consecutively for 3, 5, 7, or 10 steps. Therefore, it can be considered crucial to apply strength exercises for jumpers and sprinters. During the STJ analysis, researchers reported a correlation of r = 0.97 (p < 0.05) with the 60 m and 200 m and a perfect correlation r = 1.00 (p < 0.05) with the 100 m [49]. Although others reported different results of the correlations of multiple jumps for shorter distances [52], this exercise constitutes a primary reference in the preparation base of these two events groups. The STJ is also called multiple jumps or sprint bounding [53]. The STJ is performed differently by jumpers as they seek more elevation of the centre of mass, while the GCT is longer. In contrast, sprinters seek to reduce the GCT and the oscillations of the centre of mass in the vertical axis with a faster projection to the horizontal axis. Through multiple jumps, the athletes can train higher horizontal propulsive forces [54] compared to exercises that mainly train the vertical application of force (51).

Our STJ distance results were superior to those found in previous studies [13, 30, 52, 54]. G-RT1 and G-PT2 also present significant improvements compared to the pretest, but G-RT1 presents a higher ES at the end of program 1. After the crossover, G-PT2, which trained PTP based on the IRSI, showed an ES twice as high as G-RT1. This could indicate that a particular volume of general jumps may be beneficial before training at more specific FHs while concentrating on maintaining the maximum intensity of the IRSI. We speculate that IRSI-based PTP stimulates connective tissue and fibers properties that ensure elastic energy storage [7]. Thus, we can infer that the enhancement of elastic energy induced by PTP positively affected the performance in this exercise.

A strong consensus is that plyometric exercises performed mainly on the vertical axis positively affect sporting activities developed with a vertical force orientation [29]. This reasonable criterion justifies why G-RT1 and G-PT2 present significant improvements at the end of programs 1 and 2. A deeper analysis of the group-time interaction allows us to understand, for example, that in DJ30, both groups present significant differences compared to the pretest, yet G-RT1 presents ES twice as high as G-PT2. This result replicated at other FHs (DJ40, DJ50, and DJ60). At the end of program 2, we can no longer observe this doubled increase in the G-RT1, but we report better ES for the G-PT2. After the end of program 1, the results were superior to those reported previously using a variety of jumping tasks [22].

In our study, at the end of program 1, the training volume was higher G-PT2 compared to G-PT1. However, after crossover, the total research volume was similar between both groups. Previous research [13] showed that, regardless of the different training volumes, the researcher reported no differences between the high-volume and low-volume groups for SLJ, STJ and sprints. This report contrasts with our results. In our study, it may be that daily control of performance variables and individualization of drop height are responsible for the significant differences and high effect sizes. A previous study investigating training volume recommended that to maximize the likelihood of significantly higher improvements in performance, one should train for more than 10 weeks, with 20 sessions and more than 50 jumps per session [55]. Our results also contrast with these criteria, and more so when the FH represents the optimal height of the athletes. The results of this study are in line with previous research recommending individualized training volumes [24]

This study is not without limitations, although sex differences have been reported to exist for reactive strength variables, [56] our study did not include female participants. This was due to the small population of this gender in the groups analyzed, so our results cannot be generalized to females. In addition, only a sample of national sprint and jumping athletes was used for the intervention, so it is recommended to investigate these results in other athletic competitive levels and sports modalities. Further research is needed to investigate the effects of IRSI on the female population. Furthermore, only a minimal set of performance markers was analyzed. Future research can analyze its effects on other markers such as power output, ground reaction force, RFD, etc.

## CONCLUSIONS

This study represents the first approach to prescribing plyometric training loads based on the IRSI. The results indicate that the IRSI significantly improved the spatiotemporal variables analyzed (SLJ, STJ, 60 m, and DJ). Overall, based on the effect size (ES), the IRSIbased PTP induced more pronounced effects on most variables. Therefore, coaches and athletes can benefit from the IRSI-based PTP as a practical marker for the training of national sprinters and jumpers. An essential aspect of this training marker is the optimization of time achieved through a significant reduction in volume based on interindividual load control. Its main Practical applications are I) IRSI reports the dynamic and gradual increase in reactive strength as a function of drop height; II) it allows for individualized monitoring of exercise volume and intensity; III) the increase in the training volume is based on the athletes’ interindividual characteristics rather than a rigid criterion set by the coach; IV) monitoring the IRSI helps identify when athletes exhibit effects on reactive strength variables. Our results also suggest that using the IRSI-based program in the drop jumps training may yield better benefits. For this program, we used intensities exceeding 95%; however, future PTPs may explore whether interventions between 90% and 100%, or various combinations of intensities, could be more effective in improving these performanceassociated variables. These findings are practical and fundamental for coaches who intend to develop plyometric training programs to enhance their athletes’ speed and reactive strength abilities.
